# DoOPSearch: a web-based tool for finding and analysing common conserved motifs in the promoter regions of different chordate and plant genes

**DOI:** 10.1186/1471-2105-10-S6-S6

**Published:** 2009-06-16

**Authors:** Endre Sebestyén, Tibor Nagy, Sándor Suhai, Endre Barta

**Affiliations:** 1 Agricultural Research Institute of the Hungarian Academy of Sciences, Martonvásár, Brunszvik u. 2., H-2462, Hungary; 2 Bioinformatics Group, Agricultural Biotechnology Center, Gödöllõ, Szent-Györgyi Albert u. 4., H-2100, Hungary; 3 Department of Molecular Biophysics (B020), German Cancer Research Center (DKFZ) Im Neuenheimer Feld 580, D-69120 Heidelberg, Germany; 4 Apoptosis and Genomics Research Group of the Hungarian Academy of Sciences, Research Center for Molecular Medicine, Medical and Health Science Center, University of Debrecen, Debrecen, Hungary

## Abstract

**Background:**

The comparative genomic analysis of a large number of orthologous promoter regions of the chordate and plant genes from the DoOP databases shows thousands of conserved motifs. Most of these motifs differ from any known transcription factor binding site (TFBS). To identify common conserved motifs, we need a specific tool to be able to search amongst them. Since conserved motifs from the DoOP databases are linked to genes, the result of such a search can give a list of genes that are potentially regulated by the same transcription factor(s).

**Results:**

We have developed a new tool called DoOPSearch  for the analysis of the conserved motifs in the promoter regions of chordate or plant genes. We used the orthologous promoters of the DoOP database to extract thousands of conserved motifs from different taxonomic groups. The advantage of this approach is that different sets of conserved motifs might be found depending on how broad the taxonomic coverage of the underlying orthologous promoter sequence collection is (consider e.g. primates vs. mammals or *Brassicaceae *vs. *Viridiplantae*). The DoOPSearch tool allows the users to search these motif collections or the promoter regions of DoOP with user supplied query sequences or any of the conserved motifs from the DoOP database. To find overrepresented gene ontologies, the gene lists obtained can be analysed further using a modified version of the GeneMerge program.

**Conclusion:**

We present here a comparative genomics based promoter analysis tool. Our system is based on a unique collection of conserved promoter motifs characteristic of different taxonomic groups. We offer both a command line and a web-based tool for searching in these motif collections using user specified queries. These can be either short promoter sequences or consensus sequences of known transcription factor binding sites. The GeneMerge analysis of the search results allows the user to identify statistically overrepresented Gene Ontology terms that might provide a clue on the function of the motifs and genes.

## Background

The traditional bioinformatics approach for transcription factor binding site (TFBS) discovery uses collections of known (experimentally verified) TFBS sequences to find their occurrences in a promoter. These sequences are usually described by a consensus sequence or a position specific matrix (PSM). There are several different databases, whose main goal is to collect these consensus sequences and matrices. Two of the better known collections are the TRANSFAC [1] and JASPAR [2] databases. They contain information for a large number of different TFBS groups. Although the consensus sequence and matrix-based approach has been used for almost twenty years [3], it still has fundamental problems, such as having either too many false positives or false negatives.

Another approach is to use sophisticated computer algorithms designed for the *ab initio *prediction of overrepresented sequence motifs in a collection of promoter sequences [4,5]. In this case the user provides a number of promoter regions, thought to be co-regulated or co-expressed, and the computer algorithm identifies statistically overrepresented sequence oligos, putative TFBSs. As the TFBSs are usually short, the promoter regions long, and the bases can vary in certain positions, there are similar problems, as in the method mentioned previously.

Besides the traditional analysis methods, the motif discovery algorithms can also be used to find possible TFBSs in the promoter regions of homologous gene sets. This method is called phylogenetic footprinting [6], and an important prerequisite of it is to collect as many orthologous promoter sequences as possible [7]. These sequences are available in public DNA databases or in broadly known genome browsers like ENSEMBL [8] or UCSC [9]. The most simple way to collect them however, is to use an orthologous promoter database, such as OMGProm [10] for the promoters of mammals, or DoOP [11] for the promoters of chordates and plants. Recent developments in comparative genomics allow us to search for conserved motifs in the full genome of a large number of species. The first collections of conserved sequence motifs are now available for downloading and/or browsing. Xie *et al*. [12] analysed the human, mouse, rat and dog whole genome alignments, and described 174 sequence motifs that were highly conserved. These motifs are now part of the JASPAR database [2], thereby they can be used in a promoter analysis. The cisRED [13] database and the CORG [14] framework are based on the human genome annotation from ENSEMBL and on the COMPARA database respectively, complemented by other sources. The extracted homologous gene sets in the cisRED database were analysed using different motif discovery programs in order to find conserved sequence regions. The DoOP database [11,15] contains more than one million different conserved sequence motifs from the promoters of chordate and plant homologous gene sets. These motifs are derived from the conserved regions of DIALIGN [16] alignments of the orthologous promoters and thus represent a collinear set of possible TFBSs.

There are a growing number of methods and websites that offer promoter analysis tools and use different combinations of the abovementioned approaches. In general, they are more or less relying on the extra information coming from comparative genomics methods, and orthologous promoter collections [17-19].

Our aim was to develop a collection of web-based tools to search with known or *de novo *discovered TFBSs, as well as with longer promoter sequences, in order to collect and examine in detail genes with similar conserved motifs in their promoter regions. In this paper we describe the DoOPSearch tool that provides a new and unique method to search the motif collections of the DoOP database with a new program called MOFEXT. The server also provides a simple pattern search, based on FUZZNUC, in the promoter regions of chordate and plant genes. To analyse the Gene Ontology terms associated with a given gene in the results, we integrated the GeneMerge program into DoOPSearch. In addition, a Perl API called Bio::DOOP was also developed for the easy manipulation and querying of the DoOP database content.

## Methods

### Determining conserved motifs from orthologous promoters of different taxonomic groups

**Figure 1:**
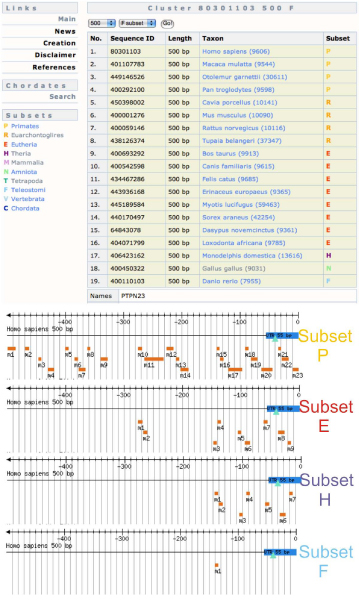
**Different number of conserved motifs from different taxonomic groups**. The PTPN23 (protein tyrosine phosphatase, non-receptor type 23) promoter cluster contains sequences from 19 different species. If the multiple alignment is made from all the sequences (subset F) we only find one conserved motif (m1). If we narrow down the taxonomic group to Theria (subset T), Eutheria (subset E) or Primates (subset P), we find 7, 9 and 23 conserved motifs respectively. The screenshots have been taken from the 500 base pair promoter cluster pages of the PTPN23 gene in the DoOP database.

In the original DoOP database [11], we used all the available orthologous promoter sequences of each gene to generate the multiple alignments and to determine the evolutionary conserved motifs. Now we are using four and ten different taxonomic categories in the chordate and plant collections when selecting sequences for the multiple alignments (Figure 1). The narrowest category amongst the plants is the *Brassicaceae *family (Table 1), which includes the Brassica species besides *Arabidopsis thaliana *(as the originating species of the orthologous clustering process). The following is the eudicotyledons category, which also includes additional dicot species like *Medicago truncatula*, *Solanum lycopersicum*, *Populus trichocarpa*, *Ricinus communis *and others. The next one is the *Magnoliophyta*, which includes the monocot species like *Oryza sativa *and *Zea mays*, and finally the *Viridiplantae*, which incorporates every species involved in the analysis. We have defined ten taxonomic categories in the chordate section (Table 2). The first one is the *Primates*, which contains the *Homo sapiens *as the originating species and all the monkeys. The following is the *Euarchontoglires*, which contains the *Primates *and rodent species like the mouse and rat. The next category is the *Eutheria *with all the placental mammals. The *Theria *category also includes the marsupials like the opossum, and the mammalian category contains all the mammals including the egg-laying platypus. The subsequent categories are the *Amniota *(mammals as well as birds and reptiles), the *Tetrapoda *(*Amniota *together with the amphibians like the Xenopus species). The *Teleostomi *category also covers the fishes like the fugu, zebrafish or *Tetraodon nigroviridis*. We also have a category for the *Vertebrate *and *Chordate *taxonomical groups, but due to the drawbacks of our orthologous finding algorithm [11], they contain only a small number of conserved motifs and are practically unusable.

**Table 1:** Number of the different taxonomic groups in the plant database

**Promoter size**	**500**	**1000**	**3000**
**Brassicaceae**	8207	4518	4257
**eudicotyledons**	2324	2263	2204
**Magnoliophyta**	370	277	267
**Virdiplantae**	41	27	21

The data is taken from the DoOP database. The description of the taxonomic groups is written in the text. The method for the generation of the taxonomic groups and conserved motifs is detailed on the DoOP database web page. .

**Table 2:** Number of the different taxonomic groups in the chordate database

**Promoter size**	**500**	**1000**	**3000**
**Primates**	21164	20484	18523
**Euarchontoglires**	14391	13665	12240
**Eutheria**	15411	14780	13744
**Theria**	4572	4390	4252
**Mammalia**	47	43	34
**Amniota**	1508	1373	1213
**Tetrapoda**	827	810	753
**Teleostomi**	1208	1161	1124
**Vertebrata**	9	2	1
**Chordata**	30	28	27

The data is taken from the DoOP database. The description of the taxonomic groups is written in the text. The method for the generation of the taxonomic groups and conserved motifs is detailed on the DoOP database web page. .

### Searching in the conserved motif collections and promoter sequences

As a result of the orthologous promoter analysis, several motif collections were defined, which consist of 6–50 base pair long consensus sequences. We developed a simple new program called MOFEXT (MOtiF sEarch and EXTension) to perform fast gapless searches in the motif collections containing even more than a million of consensus sequences.

As input, the MOFEXT program requires one or more query sequences and one or more motif lists. The program slides a given length of window specified by the user, over the query sequence, and starts to compare it with the first consensus motif from the motif collections. Sliding the window over the consensus motif, the program calculates a score for each position using a predefined scoring matrix. The overall score is then calculated by adding the individual scores of each position together. The program also calculates the maximum possible score for each window of the query sequence by comparing it with itself. Using these scores, the program calculates a percentage value for each pair of query and motif sequence window. If this percentage value is larger than the cut-off value, and the length of the query and the consensus motif from the list is longer than the window size, the program tries to extend the match in both directions. This means that the program calculates the score as above for each possible overlapping window with an increasing size between the query and consensus sequence, and keeps the one with the highest score. The MOFEXT program accepts the standard IUPAC nucleotide.

For the search in the sequences of individual promoter regions, the FUZZNUC program was used from the European Molecular Biology Open Software Suite (EMBOSS) [20].

### Developing a Perl API for handling and analysing the DoOP data

In order to allow simple access of the data available in the DoOP database we have developed a set of Perl modules called Bio::DOOP. These modules are following the style of the BIOPERL modules [21], and used mainly for querying the MySQL backend of the DoOP database. The Bio::DOOP modules are available from the CPAN Perl repository  with a complete documentation. They are suitable for using in individual PERL scripts for querying the DoOP database through the net, and for building more complex promoter analysis tools. It is possible for example to simply download species-specific promoter sequences, determine the positions of conserved motifs, or to download the conserved motif sequences. The Bio::DOOP Perl modules also run and parse the results of the MOFEXT program, the FUZZNUC program from the EMBOSS package [20], and the GeneMerge Perl script [22].

### Construction of the DoOPSearch tool

The DoOPSearch tool is running on a two processor Linux server. The forms are generated using PHP scripts. The programs are called and their results processed using Perl scripts. Most of the data, including the search results, are stored in a MySQL database. The reading and writing of the MySQL database is carried out with the Bio::DOOP Perl modules. The standalone MOFEXT program was written in standard C language. It is available upon request as source code or in precompiled binaries for different platforms. A typical MOFEXT or FUZZNUC search with default parameters ranges in about 10–20 seconds. Depending on the input data and search parameters, however, running a query and processing the results can take several minutes or up to an hour. The database design is basically the same as in the DoOP database, with an additional motif search layer, which can't be implemented with MySQL queries [11], and provides a simple and easy to use interface for searching, browsing and analysing the results [15].

## Results

The DoOPSearch tool provides an easy to use interface for searching in the promoter data provided by the chordate and plant sections of the DoOP database. The searches result in lists of genes, containing similar sequences to the query in their promoter regions. These gene lists can be further analysed with the GeneMerge program to discover statistically overrepresented Gene Ontology terms [23]. The first step of the workflow is similar to that of the DoOP database. After choosing the chordate or plant section, the user can enter different queries and parameters for searching in the conserved motif collections and promoter sequences. The query types and parameters together with the results returned will be described in detail in the following sections.

### Searching in the conserved motif collections

The MOFEXT search is suitable for searching in the different conserved motif collections. The input sequence can be a consensus sequence motif or part of a promoter/UTR sequence. The purpose of this type of search is to find genes sharing the same putative TFBSs or promoter fragments. Finding a similar sequence in a conserved position can be a strong indication of some kind of biological function. The sensitivity and specificity of the search depends on the parameters used. The word size parameter heavily influences the sensitivity and speed of the initial search. It is worth considering using a large word size, or even the whole length of the motif if the query is a known TFBS, and set a rather low cut-off value. In the case of smaller word sizes, the number of hits will increase dramatically, as the MOFEXT program applies the cut-off value prior to extending the hit motif sequence and the results will contain a large number of similar but small motifs. After testing several alternatives the EDNAFULL scoring matrix was chosen from the EMBOSS package [20]. The consensus sequences from the DoOP database are post-processed and divided into different sub-collections of motifs. The DoOP database for example, contains orthologous promoters with three different sizes (500, 1000 or 3000 base pair length), and so the motifs fall into three different category according to the length of the originating promoters. It is also possible to pick motif collections with the consensus generated using promoter sequences only from a strictly defined monophyletic taxonomic group (described in the Methods section). Users can combine the available motif collections, but in the case of larger collections, the running time increases significantly.

The results include the cluster id of the promoter cluster containing the motif similar to the query, a short description, the type and size of the cluster, and the score of the motif. The results are sorted by the MOFEXT scores, the cluster containing the motif with the highest score coming first. It is also possible to sort the results by cluster id or filter them by the score value, cluster type or size. Furthermore, the sequences can be downloaded from here in FASTA format, and a GeneMerge analysis (described later) can be launched.

### Searching in the full promoter regions

The FUZZNUC search tool utilizes the FUZZNUC program of the European Molecular Biology Open Software Suite (EMBOSS). The users can choose between the 500, 1000 and 3000 base pair promoter sets. There are also options to search only in the promoter sequences of a given reference organism (*Homo sapiens *in the case of chordates and *Arabidopsis thaliana *in the case of plants), or every sequence from different species available in each cluster of promoters. The result page is similar to the MOFEXT result page with the same options, such as observing a given promoter cluster, sorting or filtering the results, downloading the sequences or launching a GeneMerge analysis.

### Gene Ontology analysis of the gene lists

If we use a single TFBS as the query in a MOFEXT or FUZZNUC search, we can assume that the genes containing the same putative TFBS in their promoter regions are under similar transcriptional control, and thus their function can also be similar. Based on this approach the gene lists can be analysed in a way similar to those coming from microarray experiments. Since most of these genes are associated with one or more Gene Ontology (GO) term [23], we can test the gene lists with different statistical methods to find over-represented GO categories. We have chosen the GeneMerge program [22] to do this on our DoOPSearch website. As the original GeneMerge program proved to be quite slow, we slightly modified it, to make it usable in our DoOPSearch tool.

The result of the statistical analysis greatly depends on the size of the input lists. In our case, both the population, and the study size can vary between different runs. The population size depends on the motif collection subsets or promoter sequence collections used in the original MOFEXT or FUZZNUC search. The study size can be altered by selecting and using different number of genes on the MOFEXT or FUZZNUC search result page. Since the results of the GeneMerge runs (especially the significance of the evaluated GO terms) can vary considerably by changing the initial MOFEXT and FUZZNUC search parameters and/or the number of genes fed to the GeneMerge program, it is worth trying several runs and several filtering parameters to find the best result.

## Discussion

### Searching for evolutionary conserved motifs

The first motif collections of the DoOP database were generated using all the available sequences of a given promoter cluster. We noticed however, that using promoter sequences from smaller taxonomic groups improves the alignments significantly and thus can yield different conserved motifs. The evolutionary sense of this approach is the following. If for example a new TFBS had arisen in the placental mammals (*Eutheria*), it would appear as conserved motif only in an alignment made with only from the Eutherian sequences. In the other alignments, which in turn contain evolutionary more distant species, it can be a non-conserved and so unnoticed region. It must be also mentioned that different orthologous promoter clusters can have different number of species, which in turn can affect the determination of conserved motifs in our algorithm. We defined the taxonomic categories based on this logic. To our knowledge there are no similar motif collections available, taking into account the evolutionary distance between the species used in the phylogenetic footprinting.

### Case studies

Basically two types of analysis can be carried out with the DoOPSearch tool. Either a longer promoter fragment, or a short conserved motif, known TFBS can be used as a query in the MOFEXT or FUZZNUC search.

**Figure 2:**
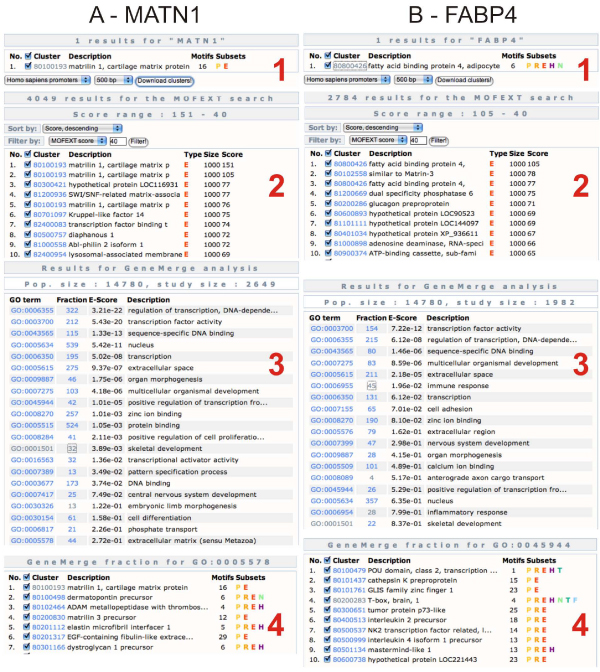
**MOFEXT and GeneMerge analysis of the 300 base pair upstream region of the *matrilin-1 *and the *FABP4 *genes**. We downloaded the 500 base pair promoter region of the *matrilin-1 *(A1) and *FABP4 *(B1) genes. We used the last 300 base pair of these sequences as a query in the MOFEXT search with the following parameters: wordsize: 8, cutoff: 70 and the 1000 base pair E subset (A2 and B2). After the MOFEXT search we got 30548 (*MATN1*) and 23463 (*FABP4*) hits. We used the score range 151-40 (*MATN1*) and 105-40 (FABP4) for the GeneMerge analysis (A3 and B3). The genes in the GO term "Extracellular matrix (sensu metazoan)" are listed in the panel A4. Some genes in the GO term "positive regulation of transcription from RNA polymerase II promoter" are listed in the panel B4.

First we demonstrate how a longer sequence can be used for promoter analysis (Figure 2). Earlier we determined both with experimental and *in silico *analysis tools, a promoter element in the upstream region of the *matrilin-1 *gene [24]. In this example we used a 300 base pair upstream fragment starting from the ATG start codon of the human *matrilin-1 *gene available at the DoOP database [15]. As a control, we chose the *FABP4 *gene and used the same promoter region. After the MOFEXT search with exactly the same parameters, we filtered the result and ran the GeneMerge analysis. It is clear that there are specific Gene Ontology categories overrepresented in each example. It is also obvious that some categories like "transcription" or "transcription factor activity" can be found in both results. The explanation for this can be that the transcription factors contain more conserved motifs in their promoter regions than other type of genes, but to confirm this, we need to perform other analyses.

**Figure 3:**
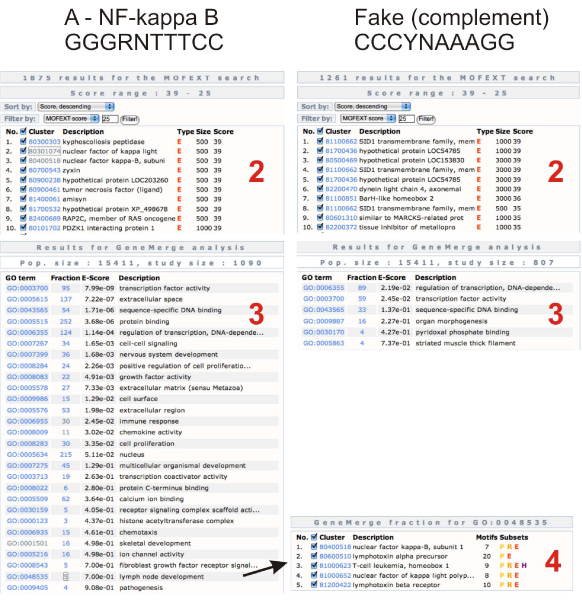
**MOFEXT and GeneMerge analysis of the *NF-kappa B *binding site**. On the left side (1) we used the *NF-kappa B *binding site consensus (GGGRNTTTCC, where R is A or G, and N is any base). On the right side we used the exact complement of the previous site (CCCYNAAAGG, where Y is C or T). We used the same parameters for the MOFEXT search in both cases: wordsize: 7, cutoff: 70 and the subset "All promoters, subset E" (A2 and B2). After the MOFEXT search we got 10697 (*NF-kappa B*) and 9303 (fake site) hits. We used the score range 39-25 in both cases for the GeneMerge analysis. At the *NF-kappa B *site, the genes from the GO category "lymph node developments" are listed.

In the other case study (Figure 3) we used the binding site of the *NF-kappa B *gene [25]. As a control we used the (not reverse) complement of the *NF-kappa B *binding site. The results clearly show the strength of our approach. Although there are some significantly enriched GO terms at the fake site as well, the difference proves the value of our analysis.

These examples show how can the DoOPSearch tool help the *in silico *annotation of longer promoter regions, known TFBSs or conserved motifs with unknown functions. It must be mentioned however, that to reach these result we had to try different parameters both in the MOFEXT search (word size, cut-off value and motif collection), and also in the GeneMerge analysis.

## Conclusion

The DoOPSearch tool in combination with the Bio::DoOP Perl modules and the MOFEXT program are suitable to search for conserved promoter motifs or sequence patterns. Although there are a number of tools and services available for computational analysis of transcriptional regulation using different methods, to our knowledge DoOPSearch is the first tool which gives the opportunity to search in chordate or plant conserved motif collections. To perform such a search, we have developed a program called MOFEXT which utilizes a simple gapless word matching and scoring algorithm in order to search in a collections of conserved motifs, like our motif collections from the DoOP database.

DoOPSearch is an ideal bioinformatics tool for researchers looking for potentially co-expressed genes, or putative TFBSs in the upstream regions of different genes. Using the MOFEXT search it is relatively easy to find genes containing conserved motifs in their promoter regions similar to the query. The query can either be a short experimentally verified binding site or a promoter region with unknown TFBSs but proven regulatory functions. By recognizing that the results from the MOFEXT or FUZZNUC searches might contain co-expressed genes similar to the results of microarray experiments, we offer a unique tool to assign Gene Ontology terms and functions to a motif. The method cerainly contains a lot of ambiguity and might give false positive results, but after a detailed analysis it might be a good indicator of the role of a TFBS in gene regulation. We believe that using improved multiple alignments (with more species for example) and using more accurately defined promoter regions, our method could be improved significantly and help in the *in silico *annotation of TFBSs.

We constantly develop and update the data and the web interface. Due to the growing number of genome annotations and available genome sequences, the quality of the conserved motif collections will improve considerably. We also: (I) try to refine and improve the existing motif collections; (II) define and use new conserved motif collections; (III) implement new filtering functions, allowing the user to define a subset of genes (for example co-regulated genes from microarray experiments) used in the search; (IV) transfer the server to faster hardware and improve the speed of the MOFEXT search by preliminary runs, comparing the available motifs with each other using default parameters.

## Competing interests

The authors declare that they have no competing interests.

## Authors' contributions

ES generated the motif subsets, modified the GeneMerge script, designed the website and helped drafting the manuscript. TN wrote the MOFEXT program and the Bio::DOOP Perl modules. EB initiated and coordinated the project and wrote the manuscript. ES, TN, EB and SS were all involved in the discussion of the data analysis and assisted in writing the manuscript. All authors read and approved the final manuscript.
